# Circular bioeconomy in dairy production: Ricotta cheese exhausted whey, from a byproduct to bioproducts, a case study

**DOI:** 10.1093/af/vfaf024

**Published:** 2025-09-19

**Authors:** David Meo Zilio, Ciro Vasmara

**Affiliations:** Consiglio per la ricerca in agricoltura e l’analisi dell’economia agraria, Rome, Italy; Consiglio per la ricerca in agricoltura e l’analisi dell’economia agraria, Rome, Italy

**Keywords:** biofuels, biorefinery, cheese, RCEW, scotta, sustainability, whey

**Implications**

- Cheese production generates large amounts of cheese whey. The common ratio between cheese and cheese whey is 1:9 w/w.
- Dairy industry waste contributes to environmental pollution. However, it can also represent a valuable resource for the circular bioeconomy if properly managed.
- Protein and fat of cheese whey can be used to make “Ricotta” and other similar dairy fresh products.
- Ricotta cheese exhausted whey or “Scotta” (RCEW) is the end product of Ricotta production.
- RCEW is rich in lactose minerals and vitamins and has the potential to be usefully used in biorefinery.

## Introduction

**Figure 1.**
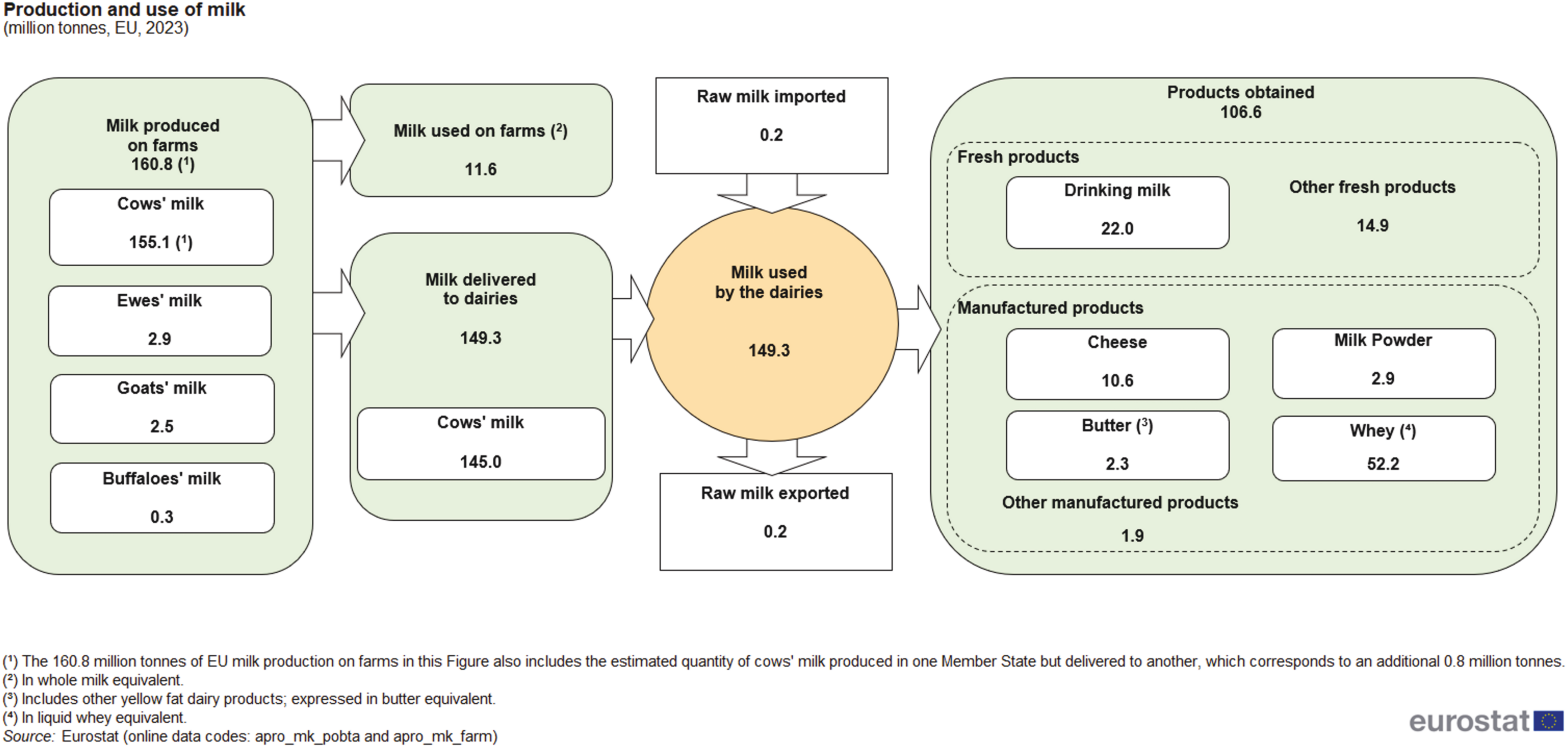
Production and use of milk in E.U. 27 (Source: https://ec.europa.eu/eurostat/statistics-explained/index.php?title=Milk_and_milk_product_statistics#:~:text=The%20EU%20also%20produced%2022.0,used%20by%20dairies%20in%202023).

The global dairy sector produced 930 million tons of milk in 2022, which was processed into a wide range of dairy products for human consumption. In 2023 (Figure 1), 160.8 million tons have been produced by EU 27, the largest global producer, followed by United States. In addition to fresh consumption, milk can be processed to produce skim milk, yogurt, cheese, cream, and buttermilk.

Some byproducts are generated during the processing of these dairy products, the main one being whey (52.2 million tons in liquid whey equivalent) from both cheese and Greek yogurt production. Cheese whey (CW) is a liquid that contains lactose, soluble proteins, lipids, minerals, and vitamins, which can be used either for human consumption or animal feeding. It is estimated that 180–190 million tons of CW are generated globally; however, currently, only 50% is used by the food and feed industry (Vasmara et al., 2021). Other residuals and waste can include products that have been downgraded or discarded. Considering their high organic content, CW and dairy wastes may represent a serious problem for the environment. In fact, the chemical oxygen demand (COD) and biochemical oxygen demand are both high. In the past, CW exceeding the industry capacity was treated in wastewater treatment plants before being discharged. As an alternative to the costly disposal, CW was proposed as a fertilizer, but many countries have strict regulations regarding its use in this manner. Currently, many dairy processors apply technologies to concentrate proteins, producing products such as whey protein concentrate, whey protein isolate, lactalbumin, and other co-products (Buchanan et al., 2023). Many of these CW-based products are used in foods, including infant formulas. Lactose is one of the major co-products obtainable from CW. Alternatively, it has been used directly or after protein extraction as cost-effective microbial fermentation medium or to produce biogas, ethanol, lactic acid, solvents, surfactants, enzymes, biopolymers, bioplastics, hormones, vitamins, and bioactive compounds (Sar et al., 2021). Whey is also used directly for producing whey cheeses like Ricotta and others. Consequently, a further liquid co-product originates from CW used for whey cheeses. Thus, CW could be considered as a raw material, whereas Ricotta Cheese Exhausted Whey (RCEW) is more distinctively a byproduct. Nevertheless, RECW still contains nutrients, mostly lactose, minerals, and some protein, available for further recycling, recovery, and reuse. There is much literature available on the use and further valorization of whey for feed, food, cosmetics, chemicals, and energy. However, there is little published research on the use of RCEW. The aim of this case study is to provide insights into RCEW and its exploitation for enhancing the circularity of the dairy sector.

## RECW Production

Even though 30–35% of the whey derived from cheese production is still discarded or used as animal feed (Garau et al., 2021), whey can be utilized for human nutrition purposes. The thermal coagulation of whey proteins produces valuable food products such as whey cheeses. Ricotta, for example, is an Italian fresh dairy product, obtained by the coagulation of whey proteins at temperatures around 80°C to 90°C with acidification, similar to cottage cheese, typically produced in United Kingdom. In Spain, Latin America, and Hispanic communities in North America, it is called Requeson (Requeijão in Brazil and Portugal) and has a coarse texture. In the United States, three types of ricotta cheese are specified: whole milk ricotta, part-skim ricotta, and ricotta, the last being obtained from skim milk, whey, or blends thereof (Farkye, 2017). In French-speaking countries, *Serac*, *Brousse*, and *Recuit* should also be mentioned. The liquid resulting from Ricotta production processes (about 90% of the whey) is RCEW and totals almost one million ton per year in Italy, where approximately 15% of CW is used to produce Ricotta (Pires et al., 2021). The mean chemical composition of CW and RCEW is reported in Table 1.

**Table 1.** Composition (% as is) of whey from cattle (various cheeses) and sheep (pecorino romano POD) milk and of RCEW

	CW^*^		RCEW^†^	
	Cow	Sheep	Cow	Sheep
Dry matter	6.61	8.20	5.67	6.42
Lactose	5.15	4.95	4.70	4.72
Protein	0.86	1.70	0.39	0.99
Minerals	0.51	0.50	0.53	0.43
Fat	0.50	2.10	0.07	0.07
Undenatured whey protein	N.A.	N.A.	0.14	0.29

^*^ Salvadori Del Prato (2001).

^†^ Mucchetti and Neviani (2006).

When compared to CW, RCEW is almost fat-free and significantly deproteinized, but the lactose content is still high enough to support further processing. Moreover, the significant organic load results in a COD up to 80 g/L (Vasmara et al., 2021), which may pose environmental risks if released into waterways, necessitating adequate treatment before disposal.

## Valorization, Current, and Potential Use of RCEW

### RCEW is a source of valuable and health-promoting food, supplements, molecules, and compounds

Historically, CW and RCEW have been fed to pigs, mostly in northern Italy, where farms are more numerous and larger. As for milk whey, there are reports on its use for laying hens (Kumar et al., 2024), broilers (Shariatmadari and Forbes, 2005), pigs, cattle and sheep (Jędrejek et al., 2016), and goats (Rapetti et al., 1995), either mixed with drinking water or feed. Given its lower nutritional value, the use of RCEW is reported to provide a poor nutritional contribution to animals (Pires et al., 2021; Fancello et al., 2024). However, considering the costs for disposal or transport associated with current waste treatment regulations and methods, RCEW valorization as a feed supplement (alone or mixed with whey) may be profitable for cheese production facilities, particularly if they also have animals or are close to livestock farms. Effects of using RCEW in monogastric and ruminant feeding are related to its high lactose content (lactose is the main nutrient for offspring and may also play an important role in adult animals, representing a source of energy for the rumen microbiome), the supply and stimulation of lactic bacteria and their role in animal nutrition (e.g., vitamin synthesis, gut and rumen health, growth promotion), and its high mineral contribution (mainly Ca and P, but also K, Mg, and Na). From a nutritional point of view, RCEW is also of interest to humans and may be used in infant nutrient formulations. Interesting properties due to its high sulfur amino acids content, which play an important role in metabolism, have been reported (Fancello et al., 2024). Bioactive peptides, which may exert antioxidant and antimicrobial effects and enhance cardiovascular wellness (Madureira et al., 2010) and glycomacropeptide, a prebiotic originated during renneting, with anti-inflammatory properties (Córdova-Dávalos et al., 2023), are also present. Other interesting components of RCEW are oligosaccharides (prebiotics) and water-soluble vitamins. Based on these characteristics, RCEW has been used to produce some functional foods and beverages, including fermented, probiotic, or fortified products (Pontonio et al., 2021). In addition, RCEW has been used for microbial starters, hyper-protein biomass, oils, and other molecules for pharmaceutical, cosmetic, or chemical applications (e.g., polymer production). An interesting application in the field of biomass production is the use of this substrate to support the growth of algae and other microorganisms (e.g., bacteria, yeasts). This approach serves a triple purpose: bioremediation of potential pollutants, production of high-value biomass with an enriched biochemical profile, and generation of numerous valuable compounds such as proteins, unsaturated fatty acids, pigments, and more. Moreover, the exhausted biomass could then be used in anaerobic digestion (AD) and other methods to reduce COD and produce energy. Stratigakis et al. (2024) obtained a significant reduction in COD, N, P, Na, and solids by cultivating *Chlorella* on CW, achieving high results in algae harvesting. Patsialou et al. (2024) used a mix of the marine microalga *Picochlorum costavermella* and the cyanobacterium *Geitlerinema* sp. to treat RCEW, confirming good biomass production and nutrient removal along with the accumulation of bioproducts. Russo et al. (2021) cultivated the red microalga *Galdieria sulphuraria* with good results, which is interesting for biorefinery applications. Other notable examples include Ribeiro et al. (2017), who employed RCEW for growing carotenoid-producing yeast; Coronas et al. (2023), who used RCEW for vitamin B_12_ production by cultivating *Propionibacterium freudenreichii*; Bosco et al. (2021) produced biodegradable polymers (polyhydroxyalkanoates [PHA]) through direct fermentation of RCEW and pretreated Toma CW using microbial mixed cultures; and Maragkoudakis et al. (2016), who used RCEW for the selective growth of lactic acid bacteria (LAB) to obtain probiotic or alcoholic (Lopes et al., 2019), fermented drinks. Finally, RCEW can be used as an additive for alfalfa silage (Mariotti et al., 2020), where it improves the fermentation performance of the silage mass.

### RCEW is a source of energy

From the perspective of circular bioeconomy, great importance must be acknowledged to biofuels. Sansonetti et al. (2009) achieved a high ethanol yield (97%) by fermenting RCEW with the yeast *Kluyveromyces marxianus*. Good results in ethanol production have also been reported by others (Vincenzi et al., 2014). RCEW was also tested as a substrate for single-cell oil (SCO) production. Such an oil can be used as biodiesel or bio lubricant as an alternative to vegetable oils (Papanikolaou and Aggelis, 2011; Shi et al., 2011). Recently, 1.83 g/L of SCO was obtained by fermenting lactose contained in RCEW at 30°C in aerobic conditions using *Lipomyces starkeyi* (Bencresciuto et al., 2024). The authors reported that these SCOs were highly resistant to oxidation, and the biodiesel technological properties met the requirements according to U.S. and EU standards and regulations. Gaseous biofuels (H₂ and CH₄) can also be produced using RCEW or RCEW permeate. High lactose fermentability and the C-to-N ratio make RCEW ideal for H₂ production in dark fermentation (DF). Unfortunately, lactose fermentation strongly lowers the pH, which is a limiting factor for H₂ yield. However, by modifying the initial pH, it was possible to enhance H₂ yield (Vasmara and Marchetti, 2017). In particular, it was observed that H₂ yield significantly increased at pH ≥ 8, reaching 3.4 mol H₂/mol lactose consumed or 1.8 mol H₂/mol hexose equivalent (the lactose molecular weight is 1.9 times higher than that of glucose). Vasmara et al. (2018) highlighted the possibility to modify the composition of the microbial community, creating a more suitable environment for hydrogen producers like Clostridia and driving DF pathways toward higher H₂ yields. Thus, the reported yield was closer to the value of 2.5 mol/mol hexose, reported for DF of other sugar-rich substrates (Vasmara et al., 2025). Further optimization of the RCEW DF process is desirable for future research activities. The drop in pH following the fermentation of lactose also significantly reduces methanogenesis. Besides, even when AD was conducted successfully, the digestate still had a high organic load incompatible with agronomic use. The possibility of exploiting the buffering effect of animal manure in co-digestion trials has been explored (Marchetti and Vasmara, 2019). Moreover, the high N content of animal manure makes it complementary with the high C content of RCEW or permeate. Thus, it would allow maintaining the C-to-N ratio at optimal values. The authors reported that the formulation of 75% RCEW and 25% pig slurry reached the highest CH₄ yield per unit of feedstock volume (10.3 mL CH₄/mL of feedstock, on average). They also found that the digestate generated as an AD end product can be used to fertilize crops for food and feed production, enhancing the circularity of RCEW.

### Main findings and future directions

**Figure 2.**
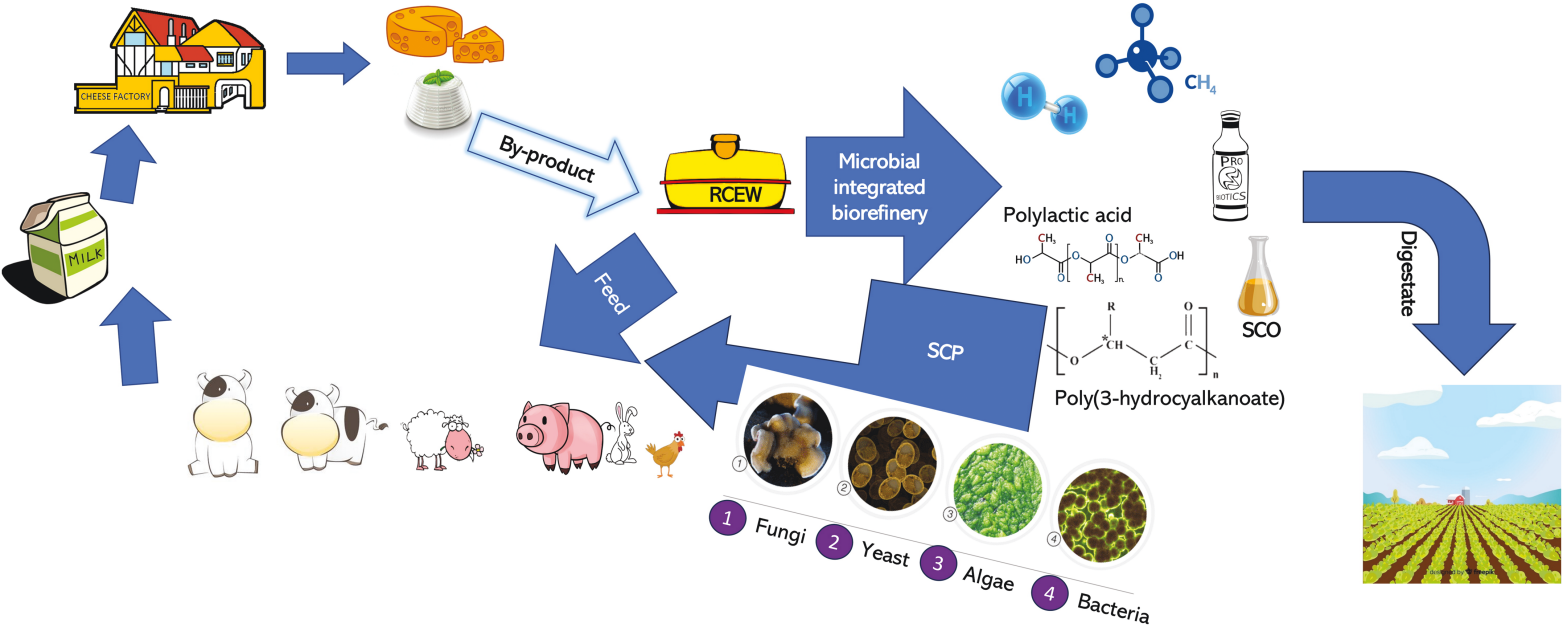
Integrated biorefinery dairy waste process.

RCEW is one of the most abundant wastewaters produced by the Italian dairy sector, but it also interests other EU and non-EU countries. From a regulatory point of view, it may be considered a waste (with limitations for agronomic purposes) and a food/feed supplement with low risk in reuse (it belongs to animal byproducts category 3, according to **Reg. (EC) No 1069/2009)**. As a waste, the disposal of RCEW represents a cost, and its reuse is worthwhile. In districts with a high concentration of livestock (e.g., in northern Italy) and presumably dairy farms nearby, using it as feed is a viable solution. However, there are some technical and technological issues to consider. Major limitations in the use of RCEW depend on its variability (species of origin, animal feeding and status, treatment and applied technology, processing plant and cheese production protocol, and other environmental factors can strongly impact RCEW quality and composition), seasonality (e.g., in Italy, the highest production occurs in spring and summer, due to cultural customs and market demand), and perishability (spoilage, oxidation, and other degradative processes may undermine safety and technological properties). Regarding this last point, which may be limiting, particularly for direct use either for humans or animals, previous experiences have shown that rapid and strong acidification to pH below 4, through inoculation with probiotic bacteria (*Lactobacillus helveticus*), results in mass stabilization suitable for direct use in calf feeding (Di Giovanni et al., 2017). The acidification power of RCEW (also rich in lactic acid) could also be used to acidify the drinking water or diet of livestock, both mammals and poultry, which has been reported to promote feed digestion and utilization, and the development of a correct intestinal microbiome (Pearlin et al., 2020). Even though RCEW has low nutritional value, its use as food and feed, also for specific categories of subjects’ diets (young, vulnerable people, athletes), may be useful. Indeed, RCEW contains energy and may improve nutrient utilization and performance thanks to lactose and other bioactive molecules and minerals. Several industrial processes are available to recover protein and peptides, sugars, fat, free fatty acids, esters, ketones, and macro-elements (clarification, ultra- and nanofiltration). Promising sectors for RCEW recycling are chemistry (carotenoids, phycocyanins, PHA, oil, and bacterial protein) and energy (biofuel and biogas production). An example of the RCEW biorefinery integrated process is illustrated in Figure 2.

RCEW can be first used as a growth medium for LAB, probiotics for the food and feed industry. Then the wastewater of LAB cultivation was exploited to produce CH₄. The digestate was eventually suitable for crop fertilization, closing the loop for the circular economy. The wastewater of LAB production can also be used to produce bioplastic, which is in turn degraded by AD (Vasmara et al., 2025). Also, in this case, the digestate can be used as fertilizer. Lastly, in an integrated biorefinery context, RCEW can be used to produce single-cell proteins (SCPs). In fact, bioplastic and oil are recovered after microbial cell destruction. The residual dead cells can then be used to produce SCP suitable as feed ingredients (Vasmara et al., 2025). Additionally, it is possible to trigger a specific amino acid formulation by adding certain trace elements to the fermentation medium. In conclusion, RCEW is a promising resource for circular bioeconomy, highlighting once again the importance of livestock in promoting circularity within agricultural systems. To achieve this, further in-depth, comprehensive, and comparative research on different and new valorization approaches, as well as integration among different sectors, is crucial. This way, it will be possible to maximize and exploit the value of dairy byproducts, save resources, and reduce the environmental footprint of human activity and livestock production.

## Conclusion

The use of CW to produce Ricotta, a valuable food, is a sustainable practice that yields RCEW, which can be variously utilized and may be a substrate for obtaining a wide range of bioproducts. Its constituents are available for industrial applications (chemistry, pharmaceutical, energy, and agriculture) either directly (i.e., as food or feed) or after recovery (filtration). This range of applications makes RCEW a resource rather than a waste. The entire system perfectly adheres to the “Food Waste Hierarchy” and “Value Pyramid” (Food➔Feed➔Industry➔Disposal).

## About the Authors

**Dr. David Meo Zilio** is a Research Scientist in the role of the Italian Council for Agricultural Research and Economics (CREA), Research Centre for Animal Production and Aquaculture. He holds a PhD in Animal Production and Products’ Quality (University of Florence, Italy) with a background in meat science, animal welfare and animal feeding and nutrition. Currently, his major interests are sustainable livestock production, methane emission measurements and reduction, and precision livestock farming. He is the Italian focal point for the Livestock Research Group of the Global Research Alliance and of the Global Methane Pledge, sector Agriculture.

**Dr. Ciro Vasmara** earned a degree in Sciences and Technologies of Animal Production from the University of Naples Federico II (Italy) and a PhD in Civil, Chemical, Environmental, and Materials Engineering from the University of Bologna (Italy). Dr. Vasmara was a microbiologist researcher at the Council for Agricultural Research and Economics (CREA). In 2019, he started a permanent position at CREA, Research Centre for Animal Production and Aquaculture, in Monterotondo, Italy. He works in the field of biogas (hydrogen and methane), biomass pretreatment, alternative biomass for bioenergy production (biodegradable plastics, spent coffee grounds, giant reed, etc.), wastewater, animal manure, chemicals and value products from biorefinery.
